# Stability studies of HIV-1 Pr55^gag^ virus-like particles made in insect cells after storage in various formulation media

**DOI:** 10.1186/1743-422X-9-210

**Published:** 2012-09-18

**Authors:** Alisson Lynch, Ann E Meyers, Anna-Lise Williamson, Edward P Rybicki

**Affiliations:** 1 Department of Molecular and Cell Biology, Faculty of Science, University of Cape Town, University Ave, Rondebosch, 7701, South Africa; 2 Institute of Infectious Diseases and Molecular Medicine, Faculty of Health Sciences, University of Cape Town, Anzio Rd, Observatory, 7925, South Africa; 3 National Health Laboratory Service, Groote Schuur Hospital, Main Rd, Observatory, 7925, South Africa

**Keywords:** Human immunodeficiency virus, Pr55^gag^, Virus-like particles, Osmolytes, Thermostability, HIV vaccine

## Abstract

**Background:**

HIV-1 Pr55^gag^ virus-like particles (VLPs) expressed by baculovirus in insect cells are considered to be a very promising HIV-1 vaccine candidate, as they have been shown to elicit broad cellular immune responses when tested in animals, particularly when used as a boost to DNA or BCG vaccines. However, it is important for the VLPs to retain their structure for them to be fully functional and effective. The medium in which the VLPs are formulated and the temperature at which they are stored are two important factors affecting their stability.

**Findings:**

We describe the screening of 3 different readily available formulation media (sorbitol, sucrose and trehalose) for their ability to stabilise HIV-1 Pr55^gag^ VLPs during prolonged storage. Transmission electron microscopy (TEM) was done on VLPs stored at two different concentrations of the media at three different temperatures (4°C, –20°C and −70°C) over different time periods, and the appearance of the VLPs was compared. VLPs stored in 15% trehalose at −70°C retained their original appearance the most effectively over a period of 12 months. VLPs stored in 5% trehalose, sorbitol or sucrose were not all intact even after 1 month storage at the temperatures tested. In addition, we showed that VLPs stored under these conditions were able to be frozen and re-thawed twice before showing changes in their appearance.

**Conclusions:**

Although the inclusion of other analytical tools are essential to validate these preliminary findings, storage in 15% trehalose at −70°C for 12 months is most effective in retaining VLP stability.

## Findings

In 2008, an estimated 1.9 million people in sub-Saharan Africa were newly infected with HIV [1] and despite advances in HIV vaccine research, a successful preventative vaccine has still not been developed [2]. While many different candidate types have been extensively investigated [3], recombinant virus-like particles (VLPs) have shown significant promise because they are non-replicating, safe [4,5] and readily recognised by the immune system due to similarity in structure to infectious virus particles. Their particulate nature also abrogates the use of adjuvants [6,7].

HIV-1 Gag polyprotein (Pr55^gag^) forms non-infectious VLPs that are morphologically similar to HIV-1 particles when expressed in isolation in insect cells [8]. We have previously shown that these VLPs significantly boost immune responses to a matched subtype C *gag* DNA inoculation in mice [9], as well as in baboons [10]. They also boost a primary vaccination of baboons with recombinant BCG expressing a subtype C HIV-1 Gag [11] and can be modified to display other proteins or epitopes, in order to stimulate a broader immune response [6]. Ye et al. [12] showed that SIV Gag-Env VLPs can boost both DNA vaccine-induced cellular and humoral responses in animals.

For VLPs to be used as vaccines, maintenance of their integrity during purification and storage is very important, as a decrease in conformational stability would affect their potency. As an example, storage temperature adversely affects the conformation of VLPs as a result of thermal instability [13,14]. The incorporation of protective molecules in the formulation buffer is thus often critical for VLP stabilisation and integrity. The cells of most living organisms produce small organic molecules known as osmolytes which stabilise and protect proteins, in response to environmental stresses [15]. Polyols (including glycerol, sucrose, sorbitol and trehalose) are found in vascular plants, fungi and algae [16]: these are natural, non-harmful substances and therefore suitable for use in vaccine formulations as stabilisers. Preliminary studies involving the use of trehalose, sucrose and glycerol as stabilisers for Norwalk [17] and rotavirus VLPs [18] and an influenza subunit vaccine [19] showed they enhance the stability of both VLPs and proteins.

This research group continually generates HIV-1 VLPs for immunogenicity studies in animals, requiring storage for varying lengths of time: accordingly, and in the interests of reproducibility in our work, in this study we evaluated the stability of HIV-1 Pr55^gag^ baculovirus-produced VLPs under different storage conditions over a period of one year. The ability of 3 readily available osmolytes (trehalose, sorbitol and sucrose) to maintain the protein in a non-aggregated state without affecting its structure, potency and function at specific temperatures was investigated. Two different concentrations of osmolytes were selected (5% and 15%) to cover the range over which stability of other VLPs has been tested [17,18]. The number of freeze-thaw cycles that a suitably formulated sample could withstand without degrading was also determined.

The HIV-1 Pr55^gag^ sequence was the same as that used for a South African HIV-1 subtype C vaccine [20,21] and originated from isolate Du422 (GenBank accession AF544010). Human codon-optimised *gag* was cloned into pFastBac (Invitrogen) under the polyhedron promoter and expressed as described [9].

Gag VLPs were produced in *Spodoptera frugiperda*-derived *Sf*9 cell suspension cultures (Invitrogen) by infecting cells with baculovirus encoding *gag* at a MOI between 2 and 10 PFU/mL. Cells were grown in SF900 serum-free insect cell medium (Gibco) supplemented with 10 μg/ml gentamycin, at 270 rpm and 27°C.

VLPs were harvested from infected *Sf*9 culture supernatant at 72 h post-infection by centrifugation (12 000 *g,* 1.5 h, Beckman SW32 Ti rotor), resuspended in 1 × PBS pH 7.4 and sedimented through a 10–50% Optiprep™ step gradient at 12 000 *g* (Beckman SW32 Ti rotor) for 3 h at 4°C. The particle band with a density of 1.17 g/ml [22] was syringe-extracted, resuspended in 1 × PBS, and re-pelleted at 12 000 *g* for 1.5 h. Purified VLPs were resuspended in 1 ml 1 × PBS.

Composition of purified Gag VLPs was confirmed by anti-p24 western blot analysis (data not shown). Purified VLPs were SDS-PAGE separated and electroblotted onto nitrocellulose (NitroBond, Osmonics Inc.) for 1.5 h at 15 V. Membranes were probed with anti-p24 rabbit antiserum (1:10 000 dilution; ARP432, NISBC Centralised Facility for AIDS reagents, MRC, UK), followed by anti-rabbit alkaline phosphatase-conjugated secondary antibody (1:5 000 dilution; Sigma) and developed with Nitroblue tetrazolium chloride/5-bromo-4-chloro-3-indolyl phosphate (Roche).

Stock solutions (60%) of sucrose and sorbitol were prepared in sterile, non-pyrogenic water for injection (SABAX, Adcock Ingram) and filtered through a 0.2 μm filter. Stock solutions (20% and 60%) of trehalose were dissolved in sterile, non-pyrogenic water at 50°C and filtered through a 0.45 μm filter. These were added to VLP aliquots in 1 × PBS resulting in final concentrations of 5% and 15% respectively, and stored at 4°C, –20°C and −70°C.

**Figure 1:**
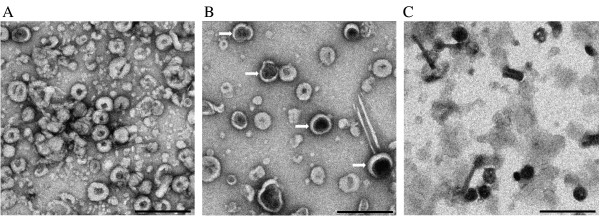
**Assessment of HIV-1 Pr55**^**Gag **^**VLPs using TEM.** (**A**) Freshly prepared VLPs resuspended in 1 × PBS. (**B**) An example where 50% or more VLPs have not retained their original appearance: VLPs retained in 1 × PBS stored at −20°C after 1 month; the white arrows indicate electron dense areas. (**C**) An example where all the VLPs (100%) have degraded: VLPs retained in 5% trehalose at 4°C after 1 month. Scale bars = 500 nm.

VLP preparations (between 1 and 2 per storage condition) were thawed and examined by TEM after 1, 3, 6 and 12 months of storage at 4°C, -20°C and −70°C. VLPs were fixed onto carbon-coated copper grids (200 mesh), stained with 2% uranyl acetate and visualized with a LEO 912 microscope. TEM (Figure 1A) confirmed the presence of Gag particles with uniform spherical shapes of 100 to 120 nm in diameter, small dark centres and dark outer membranes indicating uranyl acetate absorption, as similarly reported by others [6,9,22,23]. Some contaminating baculovirus particles and other protein aggregates were present, as previously seen in VLP preparations used for animal testing [9-11]. Integrity of between 50 and 70 VLPs was graded according to the average number of VLPs per three representative viewing frames at 10- to 15 000 × magnification which had the same appearance as when they were initially purified. A change in appearance was regarded as a change in particle size, uniform shape, or electron dense area which indicates a change in stain accumulation and hence a change in structure [23]. Changes in the VLP core [22] could result in degradation or denaturation of the CA and NC proteins that make up the Pr55^gag^ polyproteins. This could cause breakdown of the VLP structure, disrupting the highly repetitive surface epitope arrangement thereby altering the extent of the immune response [24].

As shown in Table 1, VLPs were graded as stable if, post storage, they looked morphologically and structurally similar to freshly purified VLPs (√), semi-unstable if preparations showed 50% or more degradation or alteration of VLPs (±) (Figure 1B), and completely unstable if preparations showed 100% degradation or alteration of VLPs (Figure 1C).

**Table 1:** Summary of morphological observations of VLPs under different storage conditions

**Storage time (months)**					
**Formulation medium**	**Temperature**	**1**	**3**	**6**	**12**
PBS	4°C	√	×	nd	nd
	-20°C	±	×	nd	nd
	-70°C	±	×	nd	nd
5% Trehalose	4°C	×	nd	nd	nd
	-20°C	±	×	nd	nd
	-70°C	×	nd	nd	nd
15% Trehalose	4°C	√	×	nd	nd
	-20°C	√	√	√	√
	-70°C	√	√	√	√
5% Sucrose	4°C	×	nd	nd	nd
	-20°C	×	nd	nd	nd
	-70°C	×	nd	nd	nd
15% Sucrose	4°C	√	×	nd	nd
	-20°C	√	±	×	nd
	-70	√	√	×	nd
5% Sorbitol	4°C	nd	nd	nd	nd
	-20°C	±	×	nd	nd
	-70°C	±	×	nd	nd
15% Sorbitol	4°C	4°C	nd	nd	nd
	-20°C	√	×	nd	nd
	70°C	√	±	×	nd

√: the formulated VLP micrograph is identical to that of the fresh batch.

±: 50% or more of the formulated VLPs in the micrograph have changed shape.

×: 100% of the formulated VLPs have changed shape.

nd: not determined.

**Figure 2:**
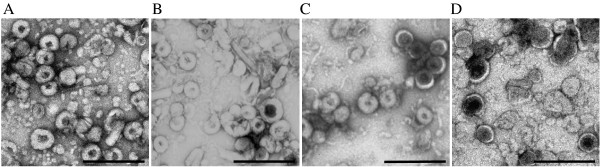
**Comparison of VLP morphology after storage in different osmolytes using TEM.** (**A**) Freshly prepared VLPs resuspended in 1 × PBS. (**B**) VLPs after 12 months storage in 15% trehalose at −70°C. (**C**) VLPs after 3 months storage in 15% sucrose at −70°C. (**D**) VLPs after 3 months storage in 15% sorbitol at −70°C. Scale bars = 500 nm.

Samples stored in 15% sucrose (Figure 2C) or trehalose were the only ones that showed no difference in VLP morphology after 3 months storage at −70°C compared to their original appearance (Table 1). However, VLPs in 15% sucrose displayed changes after 6 months at −20°C or −70°C, whereas the 15% trehalose preparations remained stable at these temperatures. At 12 months in 15% trehalose, between 10% and 40% VLPs stored at −20°C showed altered morphology (data not shown), whereas no changes were observed at −70°C (Figure 2B) compared to freshly made VLPs (Figure 2A). This corroborates work of others who used trehalose to stabilise Norwalk [17] and rotavirus VLPs [18] and influenza vaccines [19]. VLPs stored in 5 and 15% sorbitol were unstable after 3 and 6 months, respectively, when stored at −20°C or −70°C (Figure 2D).

In general, lower concentrations of osmolytes (5%) did not confer stability to VLPs at the three temperatures tested after one month. Indeed, VLPs stored in the low osmolyte concentrations at 4°C were less stable than those stored in PBS alone. While we did not investigate this specifically, 30 years experience of working in the laboratory with purified plant viruses and VLPs indicates that most preparations are more stable in buffers of ionic strength above 0.05 – and the low-osmolyte preparations were lower than this. Accordingly, we surmise that both ionic and cryoprotective agents are necessary for stabilisation. All higher percentage (15%) osmolytes tested retained the original VLP appearance at 4°C for one month, indicating they provided a provisional stabilisation medium.

**Figure 3:**
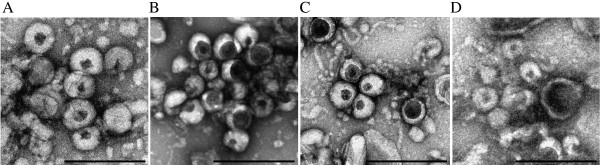
**VLPs stored in 15% trehalose at −70°C and subjected to different freeze-thaw cycles.A**) 0 freeze-thaw cycles. **B**) 1 freeze-thaw cycle after 1 month. **C**) 2 freeze thaw cycles after 3 months; white arrows indicate electron dense areas. **D**) 3 freeze-thaw cycles after 6 months. Scale bars = 500 nm.

Single aliquots of VLPs stored at −70°C in 15% trehalose were subjected to 3 freeze-thaw cycles and analysed for morphological changes using TEM. This was carried out over a period of only 10 months due to time constraints with the sample being thawed on ice for 1 h after 1, 3, 6 and 10 months (to corroborate results from the osmolyte tests above). Results showed that VLPs can withstand 2 freeze-thaw cycles, after which a change in their appearance occurred (Figure 3). After the 2^nd^ freeze-thaw cycle, over 50% of the VLPs lost their uniform spherical shape (Figure 3C).

In conclusion, this preliminary investigation into VLP storage and stability enabled us to compare the ability of sucrose, trehalose and sorbitol to stabilise HIV-1 Pr55^gag^ VLPs at three different storage temperatures (4°C, -20°C and −70°C) over the period of a year. Sorbitol and sucrose were ineffective in retaining particle stability and conformation, whereas 15% trehalose was highly effective in stabilising the VLPs for at least 1 year at −70°C. In addition, VLPs stored under these conditions could withstand up to 2 freeze-thaw cycles before exhibiting changes in their uniformity.

Our report provides valuable baseline information on exploring the long term-storage stabilisation of potential HIV-1 VLP vaccine candidates. This method is now used to store VLPs made for continued immunogenicity studies in our research group. Other techniques such as asymmetrical flow field flow fractionation (AFFFF-MALS) and electrospray differential mobility analysis (ES-DMA) [25] may provide more information on the VLP characteristics. In parallel, the ability of VLPs stored under these conditions to stimulate an appropriate immune response will need to be tested.

## Competing interests

The authors declared that they have no competing interest.

## Authors' contributions

AL produced and purified the VLPs and carried out the TEM. AM and EPR supervised the work, revised the manuscript for important intellectual content, and together with A-LW, conceived of the study, and participated in its design and coordination. All authors read and approved the final manuscript.
